# Left Ventricular Fibrosis by Cardiac Magnetic Resonance Tissue Characterization in Chronic Mitral Regurgitation Patients

**DOI:** 10.3390/jcm13133877

**Published:** 2024-07-01

**Authors:** Catalina Ileana Badau Riebel, Lucia Agoston-Coldea

**Affiliations:** 1 Department of Cardiology, “Iuliu Hatieganu” University of Medicine and Pharmacy, 400347 Cluj Napoca, Romania; luciecoldea@gmail.com; 2 Department of Internal Medicine, “Iuliu Hatieganu” University of Medicine and Pharmacy, 400347 Cluj Napoca, Romania

**Keywords:** chronic mitral regurgitation, cardiac magnetic resonance, myocardial fibrosis, T1 mapping, late gadolinium enhancement

## Abstract

**Background**: Left ventricular remodeling in chronic mitral regurgitation (MR) encompasses two types of myocardial fibrosis: replacement fibrosis, identified by late gadolinium enhancement (LGE), and diffuse interstitial fibrosis, assessed by pre- and postcontrast T1 mapping techniques. These may explain irreversible LV dysfunction after MR correction. We aimed to assess the presence of myocardial fibrosis in patients with moderate and severe MR with no criteria for surgery versus mild MR controls. **Methods**: We enrolled 137 patients with chronic primary MR and 130 controls; all underwent cardiac magnetic resonance, and were followed up in a median of 2.9 years to assess mortality and the need for mitral valve replacement. **Results**: Patients in the study group displayed significantly higher degrees of LGE (28.4% vs 7.69%, *p* < 0.05), higher native T1 values (1167 ± 58.5 versus 971 ± 51.4 (*p* < 0.05)), and higher extracellular volumes compared to controls (32.3% ± 3.5 versus 23.9 ± 2.2, (*p* < 0.05)). The composite outcome occurred in 28 patients in the study group (20.4%), and significantly higher with LGE+ (78.5%). Replacement fibrosis (HR = 1.83, 95% CI, *p* < 0.01) and interstitial fibrosis (HR = 1.61, 95% CI, *p* < 0.01) were independent predictors for the composite outcome. **Conclusions**: Patients with moderate and severe MR with no criteria for surgery still exhibit a significant degree of both replacement and interstitial fibrosis, with prognostic implications.

## 1. Introduction

Persistent left ventricular (LV) dysfunction after mitral valve surgery remains a problem in chronic mitral regurgitation (MR). Current guidelines for intervention rely on the presence of symptoms and the echocardiographic assessment of LV size and function, expressed by the left ventricular ejection fraction (LVEF) [1,2].

In chronic MR, increased volume preload of the LV results in progressive cardiac remodeling, initially by myocyte hypertrophy, to compensate for increased wall stress, followed by maladaptive remodeling, with extracellular matrix expansion. Myocardial fibrosis in these patients can occur in the absence of LV dilation and dysfunction [3]. The decline in the LVEF may reflect already irreversible myocardial damage, with persistent and progressive LV dysfunction after mitral valve repair/replacement [3]. Identifying early markers of LV dysfunction using multimodality imaging may improve prognosis by optimizing the timing of MV surgery.

Correctly assessing MR severity is paramount in the surgical decision for patients with primary chronic MR, particularly in asymptomatic patients with preserved LV systolic function. Echocardiography is the main method of assessing MR severity, integrating multiple parameters, according to an algorithm provided by the current American Society of Echocardiography guidelines [4]. However, the quantitative methods for assessments of MR severity, such as vena contracta and proximal isovelocity surface area (PISA) methods to measure effective regurgitant orifice area (EROA) and regurgitant volume (RVol), have important limitations [5,6,7,8], due to regurgitant orifice morphology, temporal changes in the regurgitant orifice, and multiple jets. Being an instantaneous calculated parameter, PISA-derived EROA does not reflect the average regurgitant orifice throughout the regurgitant phase [4], and it assumes that the PISA shape is hemispheric. This is not the case for many regurgitant jets, and this method is not feasible in cases of multiple jets.

There is also a high interobserver and intraobserver variability for many parameters, resulting in low reproducibility [5].

Cardiac magnetic resonance (CMR) is able to evaluate MR independent of the jet characteristics that limit echocardiography, by using the native contrast between the blood pool and the myocardium in the steady-state free procession (SSFP) and choosing the imaging plane independent of body status. Unlike echocardiography, CMR relies on quantitative parameters for assessing MR severity, mainly the regurgitant volume (RVol) and regurgitant fraction (RF) [4]. The RVol is usually calculated as the difference between the LV stroke volume (calculated from the short-axis LV cine stack) and the aortic forward volume (obtained from the phase velocity encoded aortic cine images). This is a reproducible method, not affected by the eccentricity, number, or direction of the regurgitant jet (Kon). CMR is thus the imaging modality of choice for the accurate assessment of MR severity, especially in patients with eccentric and multiple jets [8] CMR is also the gold standard for assessing the consequences of volume overload on the atrial and ventricular chambers size and function, using cine-SSFP short axis imaging.

Importantly, CMR allows tissue characterization using different techniques [9]. Late gadolinium enhancement (LGE) is the established method for the identification of replacement fibrosis and the characterization of myocardial scarring [10]. The presence and extent of replacement fibrosis by LGE carries prognostic implications [11,12].

In patients with chronic MR by MPV, it has been shown that LGE, mainly located at the inferolateral LV basal wall and at the level of the papillary muscles (PMs) [3,13], is partly secondary to abnormal PM traction/mechanical stress in response to increased mitral chord tension [14]. While LGE has commonly been used to detect fibrosis in chronic MR patients [15], more recently, Scatteia et al. found that a darkened appearance of PM on cine images is a typical feature of MVP [16]. Finally, the presence of interstitial fibrosis increases native T1 values, whereas postcontrast T1 values are shortened. Using the difference between pre- and postcontrast T1 values in relation to the patients’ hematocrit, the extracellular volume (ECV) can be derived [17], another parameter of prognostic value [18]. This parameter has not been described at the PM level.

Therefore, identifying the presence of fibrosis using CMR imaging techniques may refine the assessment of the impact of MR volume overload on the LV myocardium, and thus optimize the time for intervention.

The main objective of this study was to characterize LV interstitial and replacement fibrosis using CMR imaging, in a cohort of patients with moderate and severe MR who do not meet the current guideline criteria for intervention, compared to controls with mild MR. The second objective was to correlate the presence of fibrosis with clinical outcomes and the need for mitral valve intervention during follow up.

## 2. Patients and Methods

### 2.1. Study Population

We conducted a prospective, case–control study on 137 patients presenting consecutively with moderate and severe chronic primary MR and 130 controls with mild mitral regurgitation, enrolled over an interval of four years (2019–2023). The participants were recruited in two centers: the Department of Internal Medicine, “Iuliu Hatieganu” University of Medicine and Pharmacy, Cluj Napoca Romania, and “Niculae Stancioiu” Heart Institute Cluj Napoca.

The patients were included based on multiparametric echocardiographic assessment of MR severity according to current guidelines [1].

The exclusion criteria were: (1) symptomatic patients with dilated LV and depressed LVEF < 60% on CMR (who met the guidelines criteria for intervention); (2) known obstructive coronary artery disease (>50% stenosis of an epicardial coronary artery), previous myocardial infarction or percutaneous coronary intervention; (3) coexisting significant aortic valve disease (>moderate stenosis/regurgitation); (4) infiltrative or inflammatory disease (such as sarcoidosis/amyloidosis); (5) hypertrophic cardiomyopathy; (6) prior cardiac surgery; (7) active malignancy/reduced life expectancy and (8) contraindications for CMR.

Contraindications for CMR were the presence of metallic objects in the body (shrapnel, aneurysm clips, etc.), non-compatible metallic devices (MR unsafe pacemakers, insulin pumps), severe chronic renal disease, with estimated glomerular filtration rate < 30 mL/min/1.73 m and claustrophobia.

The control group consisted of 130 patients with mild MR, with similar demographic criteria to the study group.

All subjects underwent clinical assessment and symptom assessment, according to New York Heart Association (NYHA) classification; blood sampling, including full blood count, hematocrit, creatinine, NT proBNP, and galectin 3; transthoracic echocardiogram; and multiparametric CMR. Significant coronary artery disease was excluded by coronary angiogram or coronary artery computer tomography.

Demographic data—including age, gender, height, weight, medical history, current medication, biomarkers (NT proBNP, galectin 3), 12 lead ECG, echocardiographic and CMR variables, clinical outcomes of mortality, necessity for mitral valve intervention, and hospitalization for heart failure—were recorded for all patients. The median follow up was 2.9 years.

**Figure 1:**
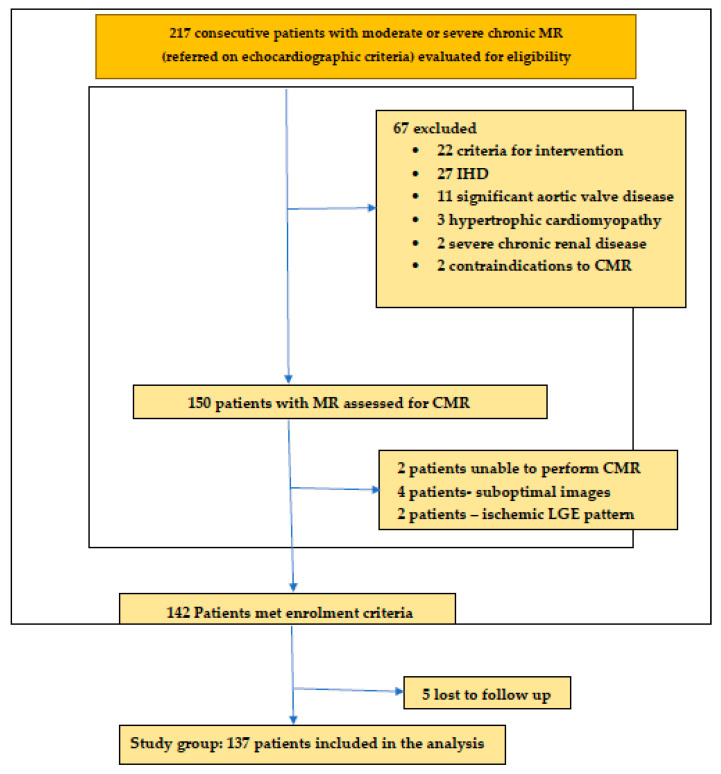
Flowchart of patient selection for the study cohort. Abbreviations: MR, mitral regurgitation; CMR, cardiac magnetic resonanceLGE, late gadolinium enhancement.

This study was approved by the local ethics committee of “Iuliu Hatieganu” University of Medicine and Pharmacy, Cluj Napoca, and was conducted in accordance with the principles of the Declaration of Helsinki. All patients were informed of the investigation protocol and signed an informed consent form (A flowchart of patient selection is presented in Figure 1. 

### 2.2. Echocardiography

Transthoracic echocardiography (TTE) was performed with a commercially available echocardiographic system (GE Vivid E90, GE Vingmed Ultrasound, Horten Norway). Evaluations of MR severity were performed by one cardiologist using standard transthoracic echocardiographic views and Doppler measurements, averaged for three cardiac cycles. MR severity was assessed as per current guidelines [19] using multiple parameters.

MV morphology was assessed visually in multiple views, with the color Doppler Nyquist limit set at 50–70 cm/s. Three components of the MR jet were identified: vena contracta (VC), proximal isovelocity surface area (PISA), and jet into the left atrium. The smallest VC distal to the regurgitant orifice, perpendicular to the direction of the jet, was measured. The Nyquist limit was decreased to 25–40 cm/s for the measurement of PISA radius at mid systole. MR peak velocity and velocity time integral (VTI) were measured using CW Doppler in apical four-chamber view. Pulmonary vein flow was interrogated using PW Doppler with the sample placed in the right upper pulmonary vein.

The criteria for mild MR were normal/mild mitral valve abnormality, small central jet with no or small convergence zone, VC width <3 mm, systolic dominant pulmonary vein flow, VTI mitral/left ventricular outflow tract (LVOT) < 1, normal LV size, normal pulmonary arterial pressure (PAP), effective regurgitant orifice area (EROA) < 20 mm^2^, regurgitant volume (RVol) < 30 mL, and regurgitant fraction (RF) < 30% [19].

Moderate MR was defined as EROA 20–39 mm^2^, RVol 30–59 mL, RF 30–49%, whereas MR with VC ≥ 7 mm, pulmonary vein systolic flow reversal, VTI mitral/LVOT ≥ 1.4, EROA ≥ 40 mm^2^, RVol ≥ 60 mL, and RF ≥ 50% were classified as severe [19].

### 2.3. CMR Imaging Protocol

Electrocardiogram (ECG) gated CMR was performed using a 1.5 T CMR system (Signa Voyager, General Electric, Boston, MA, USA) with a 33-chanel phased array surface receiver coil.

The standard CMR scanning protocol, in accordance with current international guidelines [20], consists of anatomical and functional LV assessment, using a steady-state free precession sequence in a short axis stack and 2-, 3-, and 4-chamber-long axis views (repetition time (TR) of 3.6 ms, echo time (TE) of 1.8 ms, flip angle of 60°, slice thickness of 8 mm, field of view of 360 × 270 mm, image matrix of 192 × 192 pixels, and voxel size of 1.9 × 1.9 × 8 mm).

Mitral regurgitant volume was calculated using an indirect measurement from the forward aortic flow, using phase contrast imaging, as the difference between the LV stroke volume and the aortic forward stroke volume. The regurgitant fraction (RF) was calculated as the ratio between mitral regurgitant volume and LV stroke volume.

The threshold for severe MR for regurgitant volume and fraction assessed by CMR was considered identical with echocardiography, as per the current guidelines [1].

Replacement fibrosis was assessed by late gadolinium enhancement sequences in short-axis LV stack and 2-, 3- and 4-chamber-long axes, 10–15 min after intravenous gadolinium contrast administration (Gadovist Bayer Healthcare 0.15 mmol/kg), with the inversion time chosen to null normal myocardium.

T1 mapping was performed on two short axis slices—basal and mid-left ventricular level. The ECG gated modified Look Locker inversion recovery sequence (MOLLI) was acquired pre and post contrast (15–20 min), with a 5 (3)3 sampling scheme, during end expiration breath hold. Partial voluming of blood was minimized by using a 20% offset from the endo- and epicardial borders.

Normal reference ranges for CMR parameters were considered, as previously recommended by Kawel-Boehm et al. [21] and Petersen et al. [22].

### 2.4. CMR Image Analysis

Circle CVi24 (version 5.12.1, Circle Cardiovascular Imaging, Calgary, AB, Canada) was used for image postprocessing by an experienced operator blinded to clinical and echocardiographic parameters. Left and right ventricular volumes, mass and ejection fraction were quantified on the short axis cine-SSFP stack, with the exclusion of papillary muscles. Epicardial and endocardial borders were automatically traced and manually corrected. Ventricular volumes and mass were indexed to body surface area (BSA).

The presence and location of replacement fibrosis by LGE was reported using the AHA 17 segment model, when present, in two orthogonal slices, and quantified using a signal intensity threshold over 5 standard deviations (SD) above a normal reference for normal myocardium [23].

Extracellular volume (ECV) was derived using pre- and postcontrast T1 values and hematocrit, for the six segments of the basal and mid-short axis slices of the left ventricle. The basal and midventricular slice were averaged to obtain a global ECV. Areas of nonischemic LGE pattern were included in the analysis.

### 2.5. Follow up and Clinical Outcomes

All patients were followed up through their GP, cardiologist, and by telephone interview at regular intervals (3 months). The indications for mitral valve intervention were in accordance with current ESC guidelines [1], and the decision for intervention was made by the treating cardiologist. A composite outcome of death, need for mitral valve intervention, and hospitalization for heart failure was recorded. The mean follow up was 2.9 years (IRQ 1–27 months).

### 2.6. Statistical Analysis

Statistical analysis was performed using SPPS (version 21, Statistical Package for the Social Sciences, International Business Machines Inc., New York, NY, USA), with a *p* value < 0.05 considered statistically significant.

Patient characteristics were reported as frequencies and proportions for categorical variables, or, for continuous variables, as means ± standard deviation when normally distributed, or as medians (interquartile range) otherwise. Differences across groups were determined by the Chi square or Fisher exact test for categorical variables and the Kruskal–Wallis test for continuous variables, as appropriate.

Differences between groups were compared using the log rank test. Linear regression and logistic regression were used to determine the characteristics associated with extracellular volume and regional replacement fibrosis, respectively.

Univariable survival analysis and multivariable Cox proportional hazards modeling was performed to determine the characteristics associated with the composite outcome.

## 3. Results

### 3.1. Baseline Characteristics

A total of 217 patients with chronic mitral regurgitation were enrolled from 2018 to 2022. After applying the exclusion criteria, the final study cohort included 137 patients with moderate and severe MR and 130 controls. Baseline characteristics are summarized in Table 1.

The mean age was 65.6 ± 7.1 years; 77 (56.2%) patients were male. Patients in the study group had a significantly higher prevalence of atrial fibrillation than controls and had been significantly more frequently treated with mineralocorticoid receptor antagonists and SGLT2.

### 3.2. Ventricular Structure and Function

Ecocardiographic and CMR parameters of ventricular function are summarized in Table 2.

Indexed left ventricular systolic and diastolic volumes were significantly higher in the study group versus control, although they did not meet the criteria for intervention. GLS and LV ejection fraction were significantly lower in the study group versus controls.

Left atrial volume index was significantly higher in the study group versus controls, reflecting volume overload.

Right ventricular indexed volumes were significantly increased, whereas the right ventricular ejection fraction significantly decreased in the study group.

Mitral regurgitation severity assessed by CMR reclassified 15 patients with moderate MR to severe MR, all of which presented with multiple or eccentric jets.

### 3.3. Replacement Fibrosis

**Figure 2:**
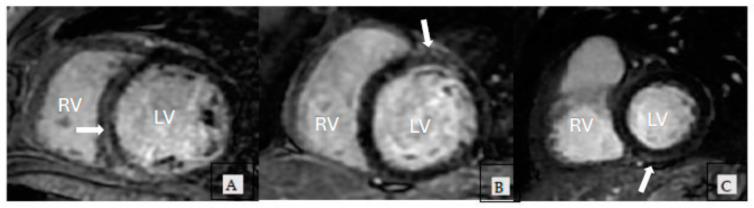
Patterns of LGE in patients with MR: Panel (**A**). Mid myocardial LGE in the basal anteroseptal LV wall (arrow) (**B**). Patchy LGE in the mid anterior LV wall (arrow) (**C**). Subepicardial LGE in the mid inferior LV wall (arrow). Abbreviations. LV, left ventricle; RV, right ventricle;MR, mitral regurgitation; LGE, late gadolinium enhancement.

Replacement fibrosis by LGE was significantly more frequent in the study group versus controls; 39 (28.4%) patients in the study group presented with various patterns of LGE versus 10 (7.69%) of controls (*p* < 0.05). Several patterns of LGE were identified—mid myocardial, patchy, subepicardial—as shown in Figure 2, the most frequent being midmyocardial fibrosis.

The presence of replacement fibrosis on LGE correlated with the regurgitant fraction measured by CMR, with a cutoff of 37%, and an increase in the LVEDVI by CMR. Regurgitant fraction measured by echocardiography did not correlate with the presence of LGE.

### 3.4. Interstitial Fibrosis

**Figure 3:**
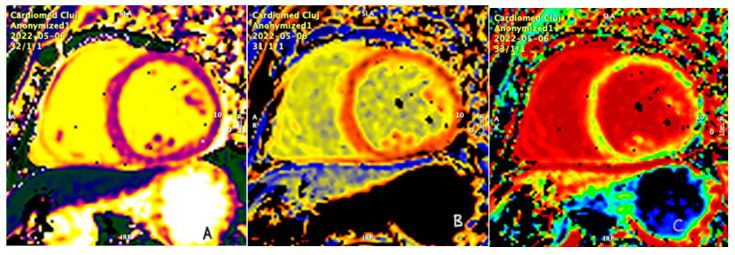
(**A**) Native T1 mapping; (**B**) postcontrast T1 mapping; (**C**) ECV map in a patient with moderate MR.

Patients in the study group had significantly higher native T1 compared with controls: 1167 ± 58.5 versus 971 ± 51.4 (*p* < 0.05). An example of a patient with moderate MR and increased native T1 and ECV is presented in Figure 3. In addition, the ECV was significantly higher in patients in the study versus controls: 32.3 ± 3.5 versus 23.9 ± 2.2 (*p* < 0.05). Both the higher native T1 values and the calculated ECV correlated with left atrial volumes measured by CMR and the regurgitant fraction measured by CMR. Neither correlated with the regurgitant fraction measured by echocardiography.

### 3.5. Clinical Outcomes and Follow Up

#### 3.5.1. Survival Analysis

During a median follow up of 2.9 years (IQR 1 to 27 months), 28 patients (20.4%), all in the study group, met the composite outcome, requiring mitral valve intervention, due to the occurrence of heart failure symptoms in 11 patients, the decrease in the LVEF in 12 patients, and the dilation of the LV in 5 patients. There were no cardiovascular deaths.

The incidence of the composite outcome was significantly higher in patients with replacement fibrosis on LGE versus patients LGE-negative in the study group (n = 22, 78.5% vs. n = 6, 21.4%, *p* < 0.05).

**Figure 4:**
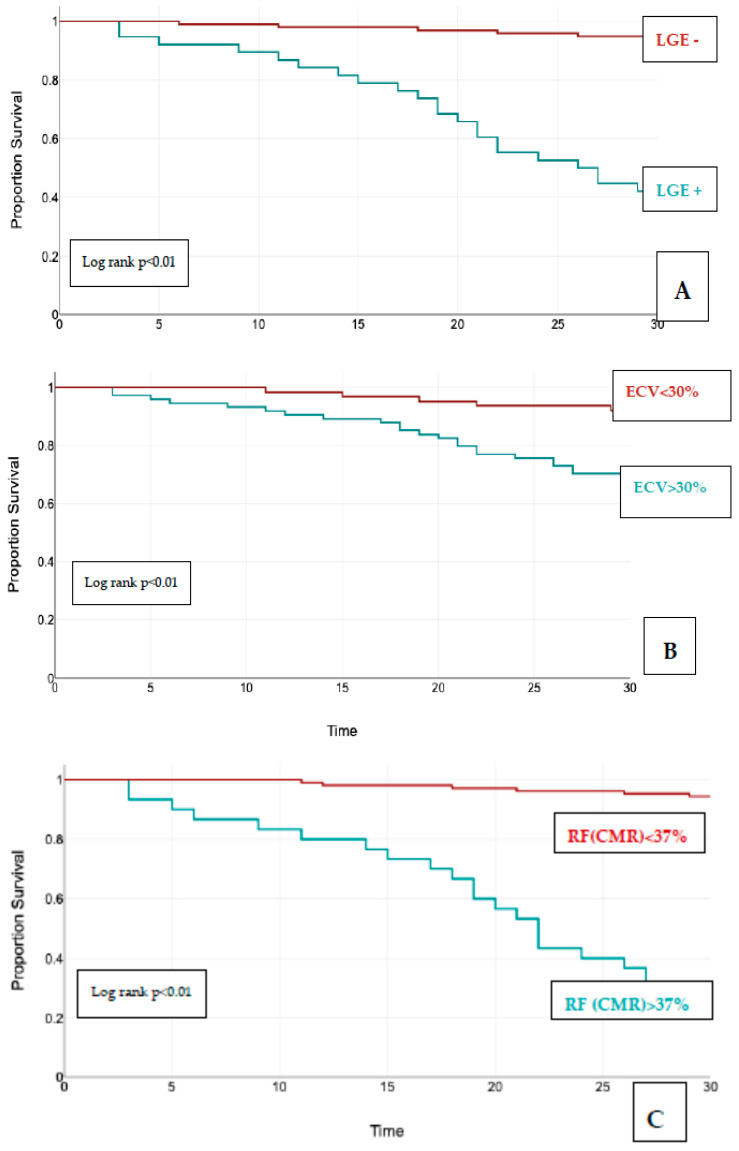

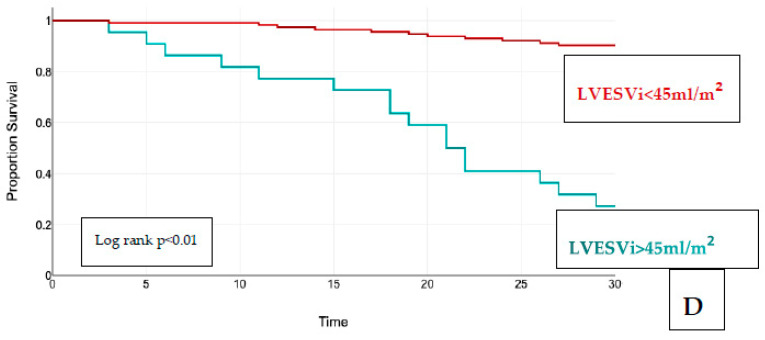
Kaplan-Meier curves for event-free survival for (**A**) LGE; (**B**) ECV; (**C**) RF; (**D**) LVESVi. Abbreviations: LGE, late gadolinium enhancement; ECV, extracellular volume; (**C**) RF CMR, regurgitant fraction measured by cardiac magnetic resonance; (**D**) LVESVi, left ventricular end systolic volume indexed.

The Kaplan–Meier curves for event-free survival showed significantly higher rates of MACEs in patients LGE+ (95% CI, *p* < 0.01), patients with increased ECV > 30% (95% CI, *p* < 0.01), increased RF > 37% (measured by CMR) (95% CI, *p* < 0.01), and an increased LVEDVi (CMR) > 45 mL/m^2^ (95% CI, *p* < 0.01) (Figure 4).

#### 3.5.2. Univariate and Multivariate Cox Analysis

Using univariate analysis and multivariate Cox regression analysis, we found two independent predictors of composite outcome: the presence of replacement fibrosis on LGE (HR = 1.83, 95% CI, *p* < 0.01) and an increase in ECV > 30% in postcontrast T1 mapping (HR = 1.61, 95% CI, *p* < 0.01).

#### 3.5.3. Risk Stratification Scoring System

**Figure 5:**
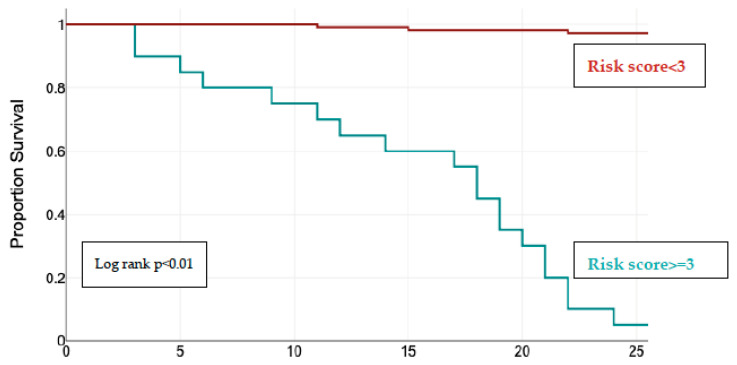
Kaplan–Meier curves for event-free survival according to risk stratification.

Combining the RF measured by CMR ≥ 37%, the LVEDi (CMR) > 45 mL/m^2^, LAVoli (CMR > 70 mL/m^2^, the presence of LGE, and ECV > 30%, we devised a risk scoring system for the composite outcome. Each of these parameters awarded 1 point. The Kaplan–Meier curve based on these five parameters showed that patients with a risk score ≥ 3 had significant higher rates of MACEs during follow up (Figure 5). Almost all composite outcome events occurred in patients with a risk score ≥ 3 (n = 26, 92%) vs. patients with a risk score < 3 (n = 2, 7%), *p* < 0.001.

## 4. Discussion

### 4.1. Replacement Fibrosis by LGE

In this prospective study, we found that patients with moderate and severe MR, who do not meet the criteria for surgery according to current guidelines, nonetheless frequently display more replacement fibrosis assessed by LGE, compared with mild MR controls. There were various patterns of LGE in our cohort, from midmyocardial to patchy and subepicardial, consistent with those reported by other studies [12].

It has been suggested by previous studies comparing patients with mitral valve prolapse (MVP) with non-MVP MR, that the underlying mechanism for replacement fibrosis in patients with MVP differs from non-MVP [24]. Patients with MVP present with a specific pattern of replacement fibrosis adjacent to the posteromedial papillary muscle that may not be related to volume overload, but to an abnormal traction and excursion of the papillary muscle towards the mitral annulus. This specific location of replacement fibrosis has been related to the risk of arrythmias in MVP patients [3,25,26].

Parameters of MR severity determined by echocardiography, mainly the regurgitant fraction (RF), did not correlate with the presence of LGE. Due to the absence of a reliable and reproducible quantitative echocardiographic parameter, the current ASE guidelines provide an algorithm for integrating multiple echocardiographic parameters to differentiate between patients with mild and definitely severe MR [4]. However, studies have shown that there is a suboptimal agreement between the ASE algorithm and CMR RVol and RF: only half of the patients with definite severe MR by the ASE algorithm had severe MR by CMR Rvol [27]. Other studies have also reported considerable discordance between echocardiography and CMR, ranging from 36% to 63% [28,29,30]. These data underline the limitations of quantitative echocardiographic parameters due to regurgitant orifice morphology and its temporal change in geometry, as well as the presence of eccentric and multiple jets.

The absence of correlation between echocardiographic parameters for MR severity and the presence of LGE in our study may be explained by an inadequate assessment of RVol and RF by echocardiography in all patients, especially in those with eccentric or multiple jets or a poor acoustic window.

On the other hand, MR severity by CMR does not directly measure the RVol and RF, thus overcoming the limitations of geometric assumptions of echocardiography. It derives these parameters by measuring the difference between the LV stroke volume (measured by SSFP imaging) and the LV forward volume (measured by phase contrast imaging).

In our study, the RF calculated by CMR, using the indirect method, correlated with the presence of LGE, with a cutoff value of 37%. This is notably lower than what the current guidelines recommend for severe MR [1], underlining the need for large cohort studies to establish CMR thresholds for MR severity. Studies reported better reproducibility and lower intraobserver variability by CMR than by transthoracic echocardiography for RVol and RF [27,31]. In a prospective study conducted by Meyerson et al., the RVol by CMR was the best predictor of which patients would develop an indication for surgery, whereas the EROA > 0.40 cm^2^ by echo was unable to differentiate the patients who would develop an indication for surgery [9].

In our cohort, the presence of replacement fibrosis on LGE predicted the need for mitral valve intervention during follow up, being an independent predictor of outcome. This is consistent with data reported by other studies, and underlines the importance of tissue characterization by CMR even for asymptomatic patients with MR.

### 4.2. Diffuse Interstitial Fibrosis

In our study, patients with moderate and severe MR had higher native T1 times and higher postcontrast calculated ECV, despite the absence of LGE and normal LVEF, reflecting significant interstitial fibrosis in this cohort.

The presence of interstitial fibrosis in the study group correlated with indexed left atrial volumes measured by CMR and with MR RF by CMR, but did not correlate with left atrial diameter and volume assessed by echocardiography, or with MR volume and fraction by echo. These findings may reflect the limitation of 2D echocardiography in patients with eccentric or multiple MR jets, where the severity of MR may be underestimated by echo criteria [31]. The eccentric remodeling of the left atrium may result in the underestimation of left atrial volumes by 2D echocardiography. Although the use of 3D echo may overcome this limitation, CMR remains the gold standard for atrial and ventricular volume assessment [32].

Interstitial fibrosis in patients with MR secondary to MVP may be influenced by more than volume overload. In patients with MVP, Pavon et al. found that the magnitude of mitral annulus disjunction (MAD) was strongly correlated with the extent of interstitial fibrosis evaluated by ECV [24,33].

It has been shown that the LV remodeling response to transcatheter mitral valve repair varies according to the magnitude of T1-mapping-evidenced fibrosis [34,35], suggesting that T1 mapping techniques may be used in follow up for the identification of patients who require early mitral valve intervention.

Although it has been shown that there is histological correlation with T1 mapping techniques [36,37,38], the specific threshold at which fibrosis is detected remains to be established [37].

The lack of standardization for T1 mapping techniques makes it difficult for these parameters to be included in a diagnostic algorithm for the workup of MR patients. However, considering the prognostic implications of interstitial fibrosis, we suggest that the presence of interstitial fibrosis in patients with MR should be assessed according to the locally validated thresholds for T1 mapping and considered in the decision making process.

### 4.3. Limitations

The study included several mechanisms for primary mitral regurgitation and did not differentiate between MVP and non-MVP. As the mechanism of fibrosis in MVP patients appears to be related to mitral annular disjunction and not necessarily the MR severity and volume overload, the presence of fibrosis in this cohort may have different implications in patient management [32].

There is a lack of standardization for T1 mapping and ECV measurements; therefore, a cutoff value for intervention is difficult to establish.

## 5. Conclusions

In patients with severe and moderate MR who do not fulfill the current guidelines criteria for mitral valve intervention, there is a significant degree of both replacement and interstitial fibrosis, demonstrated by CMR techniques, versus controls. The presence of LGE in this cohort was correlated with a higher risk for mitral valve intervention and heart failure hospitalization. By allowing tissue characterization and accurate measurements of left ventricular volumes and mitral regurgitation fraction, CMR may help stratify the risk in patients with mitral regurgitation and select patients for early mitral valve intervention in order to prevent major cardiovascular events, before irreversible left ventricular dysfunction has occurred.

Future multicenter randomized trials are needed to establish the role of fibrosis detection by CMR in the decision making process of MR management; however, this study underlines the paradigm shift that the focus in mitral valve interventions should be on the myocardium.

## Figures and Tables

**Table 1:** Baseline characteristics of patients.

Clinical Data	Study Group (n = 137)	Control Group (n = 130)	*p* Value
Age (years), mean, SD	65.6 ± 7.1	63.2 ± 11.6	NS
Male sex, n (%)	77 (56.2)	69 (53.07)	NS
Body mas index (kg/m^2^), SD	25.5, 4.8	24.7, 4.4	NS
NYHA Class I/II/III	39/98/0	33/97/0	NS
**Comorbidities**			
Hypertension, n (%)	78 (56.9)	70 (53.8)	NS
Diabetes	45 (32.8)	41 (31.5)	NS
Atrial fibrillation	24 (17.51)	12 (9.23)	0.0271
Chronic kidney disease	19 (13.86)	5 (11.53)	NS
**Blood tests**			
Hemoglobin (g/dL)	12.7 ± 1.9	12.9 ± 1.8	NS
NT-proBNP (pg/mL)	327 [171–729]	72 [31–157]	0.0037
eGFR (mL/min/1.73 m^2^), SD	77 ± 13.8	79 ± 12.5	NS
**Medications**			
ACEIs/ARBs/ARNI, n (%)	98 (71.53)	92 (70.76)	NS
β-blockers, n (%)	96 (70.07)	90 (69.23)	NS
SGLT2 inhibitors	22 (16.05)	9 (6.92)	0.0191
MRAs	21 (15.32)	12 (9.23)	0.0346
Diuretics	29 (21.16)	25 (19.23)	NS

Abbreviations: SD, standard deviation; NYHA, New York Heart Association; NT-proBNP, N terminal pro Brain Natriuretic Peptide; eGFR, estimated glomerular filtration rate; ACEI, angiotensin conversion enzyme inhibitor; ARB, angiotensin receptor blocker; ARNI, angiotensin receptor–neprilysin inhibitor; MRA, mineralocorticoid receptor antagonist; SGLT2, sodium–glucose cotransporter 2.

**Table 2:** Echocardiography and CMR parameters.

Parameters Transthoracic Echocardiography	Study Group	Control Group	*p* Value
LVEDVI (mL/m^2^)	112 ± 36.2	75.3 ± 19.7	0.0275
LVESVI (mL/m^2^)	39 ± 18.3	23 ± 11.3	0.0414
LVEF (%)	65 ± 8	68 ± 8	NS
LVSVI (mL/m^2^)	33.5 ± 14.4	23.7 ± 11.4	0.0195
GLS (%)	17 ± 3.1	20 ± 3.7	0.0360
LAVolI (mL/m^2^)	73.4 ± 25.7	32.2 ± 26.2	0.0073
TAPSE (mm)	28 ± 7	29 ± 8	NS
MR RVol (mL)	41.8 ± 12.7	13.5 ± 8.1	0.0023
MR RF (%)	39.8 ± 11.3	7.8 ± 6.4	0.0017
**CMR parameters**			
Native T1 values (ms)	1167 ± 58.5	971 ± 51.4	0.0211
Postcontrast T1 values (ms)	381 ± 26.5	437 ± 38.2	0.0192
ECV	32.3 ± 3.5	23.9 ± 2.2	0.0226
LVEDVI (mL/m^2^)	123 ± 39.5	79.1 ± 18.3	0.0061
LVESVI (mL/m^2^)	45 ± 17.8	27 ± 12.1	0.0085
LVEF (%)	61 ± 5	69 ± 4	NS
LVSVI (mL/m^2^)	30.2 ± 11.5	23.5 ± 12.2	0.0191
LV mass index (g/m^2^)	77.3 ± 24.8	69.9 ± 17.1	0.0388
RVEDVI (mL/m^2^)	76.1 ± 20.1	66.9 ± 19.0	0.0267
RVESVI (mL/m^2^)	33.0 ± 13.7	31 ± 14.4	0.0419
RVEF (%)	51.8 ± 13.5	56.0 ± 12.4	NS
RVSVI (mL/m^2^)	34.0 ± 15.7	25.7 ± 11.8	0.0381
RVol (mL)	48.7 ± 11.3	12.5 ± 7.4	0.0106
RF (%)	46.9 ± 10.2	8.5 ± 5.1	0.0043
LGE+	39	10	0.0029
PM LGE	9	1	0.0011
LAVolI (mL/m^2^)	81.5 ± 19.4	34.3 ± 18.1	0.0051

Abbreviations: CMR, cardiac magnetic resonance; LVEDVI, left ventricular end-diastolic volume index; LVESVI, left ventricular end systolic volume index; LVEF, left ventricular ejection fraction; LVSVI, left ventricular stroke volume index; GLS, global longitudinal strain; LAVolI, left atrial volume index; RVEDVI, right ventricular end-diastolic volume index; RVEF, right ventricular ejection fraction; RVESVI, right ventricular end-systolic volume index; TAPSE, tricuspid annular systolic excursion; ECV, extracellular volume; RVol, regurgitant volume, RF, regurgitant fraction; LGE, late gadolinium enhancement; PM, papillary muscles.

## Data Availability

The data presented in this study are available on request from the corresponding author.
